# Free Flap Transplantation on the repair of defects caused by oral and maxillofacial tumors resection

**DOI:** 10.12669/pjms.35.5.316

**Published:** 2019

**Authors:** Xuemei Dai, Pengchong Li, Haiyan Xu

**Affiliations:** 1 Xuemei Dai, Health Management Center, Binzhou People’s Hospital, Shandong 256600, China; 2 Pengchong Li, Stomatology Department, Binzhou People’s Hospital, Shandong 256600, China; 3 Haiyan Xu, Endoscopy Room, Binzhou People’s Hospital, Shandong 256600, China

**Keywords:** Free flap transplantation, Oral and maxillofacial tumor, Repair

## Abstract

**Objective:**

To investigate the efficacy of free flap transplantation on the repair of tissue defects after oral and maxillofacial malignant tumor resection and its effects on serum sialic acid (SA) and interleukin-2 (IL-2).

**Methods:**

Fifty-eight patients with oral and maxillofacial tumors were enrolled and set as the observation group. After the tumor resection, free flap transplantation was performed for postoperative repair. The postoperative efficacy, adverse reactions and follow-up indicators were observed. Moreover, 55 patients with benign tumors were enrolled into the control group, and 55 healthy persons were set as the healthy group. The levels of SA and IL-2 of the three groups were detected.

**Results:**

In the observation group, 55 patients were successfully repaired (94.83%); 15 patients had adverse reactions after surgery. The follow-up duration was two to four years, and 45 patients survived for three years, with a survival rate of 77.59%. Before treatment, the serum SA level of patients with oral malignant tumor was significantly higher than those of the control group and healthy group, while the IL-2 level was significantly lower than those of the other two groups, and the differences were statistically significant (P<0.05). The serum IL-2 level in the observation group one day and fourteen days after surgery was higher than that before surgery, while the serum SA level was lower than that before surgery; the differences were statistically significant (P<0.05).

**Conclusion:**

The application of free flap transplantation in the repair of postoperative tissue defects of oral and maxillofacial tumor resection is effective and has less complications, and the determination of both serum SA and IL-2 levels offers important references to recovery of patients with oral and maxillofacial tumors and prognosis evaluation.

## INTRODUCTION

Oral and maxillofacial tumor is clinically one of the most common tumors, and it mainly includes squamous cell carcinoma, glandular epithelial cancer and lymphatic epithelial cancer. According to incomplete statistics, malignant oral and maxillofacial tumors account for about 5% of systemic malignant tumors.^1^,^2^ Clinically, patients are mostly treated by surgical resection.^3^ However, in most cases there are tissue defects after surgery, and they affect the facial functions of patients, which leads to dysfunction and facial damage, and brings bad influence to the patients’ life.^4^,^5^ If the scope of surgery is limited, it will lead to an increase in tumor recurrence rate. Therefore, the tissue flap used to repair the defects immediately after the resection of the oral and maxillofacial malignant tumor can restore the local facial function and appearance of the patient, which can improve the quality of patients’ life after surgery. Previously, tissue defects after resection of maxillofacial malignant tumors was mostly repaired by delayed re-construction, prosthesis wearing, free skin transplanting, and repair of adjacent pedicle flaps, which had characteristics of long treatment duration and poor function recovery.^6^,^7^ In recent years, with the continuous progress of free skin flap transplantation and repair technology, pectoralis major myocutaneous flaps, anterior wall tissue and anterolateral femoral flaps have been gradually applied in the treatment of oral and maxillofacial defects, and moreover, some therapeutic effects have been achieved. Free tissue flaps repair technology can promote wound healing, accelerate recovery of language and masticatory functions, and significantly improve the clinical efficacy on the premise of guaranteeing the normal tissue of patients with oral and maxillofacial defects. A study reported that the success rate of the application of the free tissue flap in the repair of tissue defects after oral and maxillofacial tumor resection could reach more than 95%.^8^ Mao et al. Found that the success rate of free tissue flap transplantation was high and the complications of the donor site were significantly less than that in pedicled tissue grafting.^9^ This study focused on the repairing effect of tissue defects after oral and maxillofacial malignant tumor resection by applying free flap transplantation, providing an exact basis for improving the life quality of patients.

## METHODS

Fifty-eight patients with tissue defects caused by oral and maxillofacial malignant tumor resection and fifty-five patients with benign tumors who were admitted to Binzhou People’s hospital between August 2013 and August 2014 were randomly selected according to the principle of convenience sampling and set as the observation group and control group. The formula for the determination basis of sample size is:


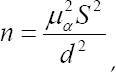


where d stands for allowable error or sampling error, S stands for standard deviation, μ stands for the fractile of standard normal distribution, and α stands for the probability of the I-th category, either unilateral or bilateral.

The patients satisfied the following inclusive criteria: having underwent histopathological biopsy or intraoperative frozen biopsy, being confirmed by postoperative conventional pathological diagnosis, having underwent preoperative clinical examination or CT, magnetic resonance and color Doppler ultrasound examination, and having underwent surgical resection only or cervical lymphadenectomy according to cervical lymph node conditions. Patients who had blood diseases, mental disorder, anemia and organic lesions in the liver, kidney, heart and lung were excluded. Moreover 55 healthy people who received physical examination of our hospital in the same period were selected. This study has approved by the ethical committee of our hospital, and all the research subjects signed informed consent.

### Treatment methods

### Surgical methods

According to the repair site and area, the flap was selected.^9^ The forearm flap was selected for 31 patients with maxillofacial and soft tissue defects in the oral cavity and tongue reconstruction, the anterior cutaneous flap or the latissimus dorsi flap was selected for 13 patients who had the defect diameter over 3.0cm, the tibial flap was selected for nine patients with the mandibular defects, and the anterior or anterior arm flap was selected for five patients with the extraoral defects. According to different recipient area, the anastomotic vessels were the superior thyroid artery, the external maxillary artery, the transverse cervical artery, the lingual artery, the anterior and posterior vein and the external jugular vein. The flaps were sutured directly with corresponding arteries and veins under the microscope, or the vessels were anastomosed with a modified 732-II vascular anastomat, and both were end-to-end anastomosis.

### Postoperative treatment

After surgery, aspirin and low molecular weight dextran were used, antibiotics were given to prevent infection, and the head was fixed for 72 hours. Within 24 hours after surgery, the color, temperature, texture and swelling of the flap were closely observed, and the blood flow in the anastomosis was detected by Doppler ultrasound. The drain tube was removed after 5 to 7 days and suture was taken out after one to two weeks.

### Observation indicators

The postoperative efficacy and adverse reactions were observed. The patients’ recurrence, swallowing and oral function recovery and survival situation were followed up. Serum sialic acid (SA) and IL-2 levels were measured. Specimen collection: venous blood drawn with an empty stomach in the morning was centrifuged at 2500 r/min for 20 minutes, and the supernatant was aspirated and stored in a refrigerator at -20 °C for testing. Elisa method was used in the M5/M5e multi-function microplate reader (Molecular Devices, USA), and the SA and IL-2 kits were supplied by Shanghai Huayi Biotechnology Co., Ltd., China.

### Statistical Analysis

Data were analysed by SPSS ver. 21.0. Measurement data were expressed as mean±SD and analyzed by paired t test, and P<0.05 meant that difference was statistically significant.

## RESULTS

There were 58 patients in the observation group, 30 males and 28 females, aged 30-72 (51.5±4.4) years, with a disease course of 1-15 (5.3±1.8) months. As to the primary sites, there were 9 cases of buccal mucosa, 14 cases of subgingival, 24 cases of tongue, and 11 cases of floor of mouth; there were 27 cases of highly differentiated squamous cell carcinoma, 4 cases of rhabdomyoma, moderately and 20 cases of poorly differentiated squamous cell carcinoma, and 7 cases of tongue root adenoid cystic carcinoma. The control group consisted of 55 patients, 23 males and 22 females, aged 32-75 (51.3±4.8) years, with a disease course of 2-18 (6.8±1.4) months. As to the primary site, there were 10 cases of buccal mucosa, 14 cases of subgingival, 22 cases of tongue, and 9 cases of floor of mouth; there were 26 cases of highly differentiated squamous cell carcinoma, 5 cases of rhabdomyoma, 18 cases of moderately and poorly differentiated squamous cell carcinoma, and 6 cases of tongue root adenoid cystic carcinoma. There were fifty-five healthy subjects, 28 males and 27 females, aged 31-74 (52.6±4.3) years. There were no significant differences in sex and age among the three groups (P>0.05), suggesting that the results were comparable.

Defects of fifty-five patients in the observation group were successfully repaired, with no scar on the face and with an efficacy rate of 94.83%, and they were satisfied with the results. There were 58 anastomotic arteries and 66 anastomotic veins, among which 50 cases were flap anastomosed with one vein and eight cases with two veins. After surgery, five patients had small area necrosis for the congestion, four patients developed vascular crisis and recovered after anti-inflammatory treatment and local treatment. Two patients had donor site infection with effusion and were healed by incision and drainage. Three patients had mild digestive system adverse reactions, such as nausea and vomiting, two days after surgery, resulting in flap vascular crisis; hence they underwent transplanted flap resection. Four patients had local hematoma which was treated by debridement and hemostasis treatment. Other flaps all survived well.

The serum SA level in the observation group was significantly higher than that in the control group and healthy groups before surgery, while the IL-2 level was significantly lower than that in the control group and healthy group (P<0.05). There was no significant difference in serum SA and IL-2 levels between the control group A and B (P>0.05, Table-I).

**Table I:** Comparison of serum SA and IL-2 levels between the observation group and the control group A and B before surgery.

Groups	IL-2 (ng/mL)	SA (μg/mL)
Observation Group (n=58)	5.15±1.38*^#^	0.81±0.31*^#^
Control Group (n=55)	16.58±4.67	0.63±0.28
Healthy group (n=55)	15.91±4.20	0.63±0.24

* ***Note:*** indicated P<0.05 compared with the control group, and # indicated P<0.05 compared with the healthy group.

The serum SA level one day and two weeks after surgery in the observation group was significantly lower than that before surgery, while the IL-2 levels were significantly increased. The difference was statistically significant (P<0.05). There was no significant difference in the serum SA and IL-2 levels one day and two weeks after surgery (P>0.05, Table-II).

**Table II:** Comparison of serum SA and IL-2 levels in the observation group before and after surgery.

Time point	IL-2 (ng/mL)	SA (μg/mL)
Before Surgery	5.15±1.38^ab^	0.81±0.31^ab^
One Day after Surgery	12.60±3.52	0.68±0.23
Two Weeks after Surgery	13.03±3.40	0.72±0.19

***Note:*** a indicated P<0.05 compare with one day after surgery, and b indicated P<0.05 compared with two weeks after surgery.

All the 58 patients were followed up successfully and the follow-up duration was two to four years (average 2.8 years). Among them, 8 patients (13.79%) had recurrence in situ, 6 patients (10.34%) had neck recurrence, and one patient (1.72%) had metastasis. Nineteen patients with tongue cancer had good swallowing function and pronunciation, five patients could have soft food, and the others all had normal diet. Eight patients died 8 months after surgery and five patients died 1.5 years after surgery because of tumor recurrence; three patients had metastasis nine months after surgery and received surgery again. Forty-five patients survived after three years, and the survival rate was 77.59%.

## DISCUSSION

In China, the incidence of oral and maxillofacial tumors keeps increasing.^10^ At present, the best treatment for this disease is surgery combined with radiotherapy or chemotherapy; however, oral and maxillofacial tumor resection causes local defects around mouth and face, which leads to dysfunction and image destruction.^11^ For patients with oral and maxillofacial malignant tumors, the corresponding tissue flaps used for defect repair immediately after resection is of great significance for the recovery of local function and appearance and the improvement of postoperative quality of life.

The author used forearm flap, anterior femoral flap, tibial flap and latissimus dorsi flap to repair the tissue defects in 58 patients after oral and maxillofacial malignant tumor resection and obtained an effective rate of 94.83%. The follow-up duration lasted for two to four years and the 3-year survival rate was 77.59%, which was similar to that of previous research.^12^ In the course of the surgery, we found that the forearm flap was easy to operate and could be used to repair strong functional tissues and organs like tongue, lip, soft palate and cheek, and be combined with flaps from other parts. The forearm flap is thin, and has unique advantages for the repair of pharyngeal and laryngeal defects,^13^ among which the priority is given to ulnar forearm flap for it has the advantages of smaller dysfunction and more beautiful appearance in the donor area.^14^ Among 58 patients in the observation group, five patients had skin flap necrosis, 2 patients local infection, 4 patients hematoma, and four patients vascular crisis. Studies have shown that the failure of free flap transplantation was mainly concerned with bad habits of patients such as smoking and drinking,^15^,^16^ systemic factors like anemia, coagulopathy and cardiopulmonary insufficiency, local factors like radiotherapy, microsurgical operations and so on. Therefore, patients should quit smoking and alcohol before surgery, and doctors should actively treat and combine diseases, correctly evaluate patients’ local conditions, standardize the operation to prevent vascular torsion, sputum, compression, and conduct postoperative monitoring to reduce the incidence of postoperative complications and repair failure rate.

Serum SA and IL-2 levels are important indicators for the measurement of oral and maxillofacial tumor resection and tissue repair. Serum IL-2 levels in patients with oral and maxillofacial malignant tumors were significantly lower than healthy persons or those with benign tumors, and IL-2 levels gradually decreased with the increase of malignancy.^17^ As an amino acid substance, SA exists on the surface of the cell membrane, and in the case of malignant or damaged cells, the SA level on the cell membrane changes significantly and can enter the blood circulation from the surface of the tumor cell; therefore, serum SA level abnormally increases in the patients with oral and maxillofacial tumors and postoperative tissues damages.^18^,^19^ In this study, the serum IL-2 level in the observation group was lower than that in the control group A and B before surgery, and the serum SA level before surgery was higher than that in the control group A and the control group B, which was consistent with the results of Shao et al.^20^ Besides, this study also found that serum SA level of patients decreased significantly one day and two weeks after treatment and IL-2 increased significantly, which suggested that free flap transplantation treatment cured the oral and maxillofacial tumors and improved tissue damage after resection.

## CONCLUSION

Free flap transplantation has an ideal repair effect on the defects caused by oral and maxillofacial tumor resection, with a low incidence of complications. It can effectively regulate the serum SA and IL-2 levels and is beneficial to the quality of life and physical and mental health. The therapy is worth promotion.

### Authors’ Contribution

**XMD:** Study design, data collection and analysis.

**XMD & PCL:** Manuscript preparation, drafting and revising.

**XMD:** Review and final approval of manuscript.
